# Supplementary material for: Prostate Apoptosis Response-4 (Par-4): A Novel Target in Pyronaridine-Induced Apoptosis in Glioblastoma (GBM) Cells
Source: Cancers (Basel). 2022 Jun 29;14(13):3198. doi: 10.3390/cancers14133198 (PMC9264948; doi:10.3390/cancers14133198)
Supplement: Supplementary file 1 [file cancers-14-03198-s001.zip › cancers 1779254-Supplementary S2.pdf]

Prostate apoptosis response-4 (Par-4): a novel target in Pyronaridine-induced apoptosis in glioblastoma (GBM) cells

Supplementary S2: Western blot raw data

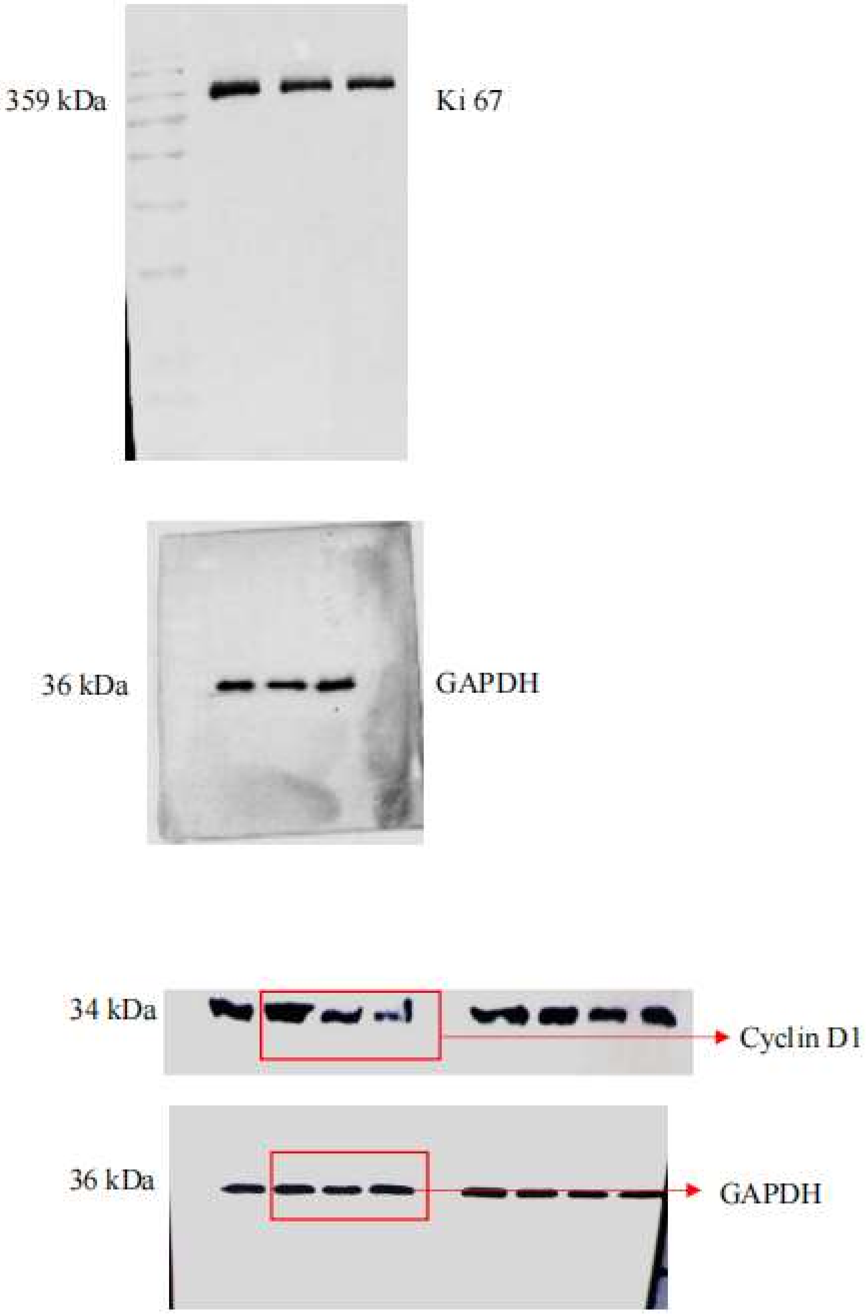

Figure S1. Raw data of Ki67, Cyclin D1 and GAPDH immunoblot from Figure 2H .

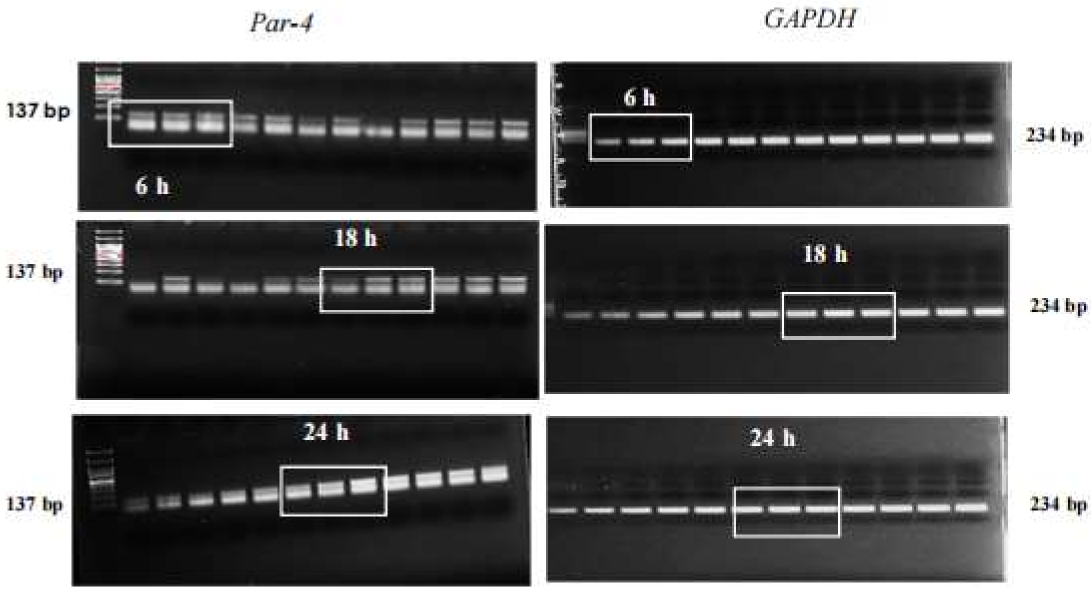

Figure S2. Raw data for agarose gel of Figure 7A Figure S2. Raw data for agarose gel of Figure 7A

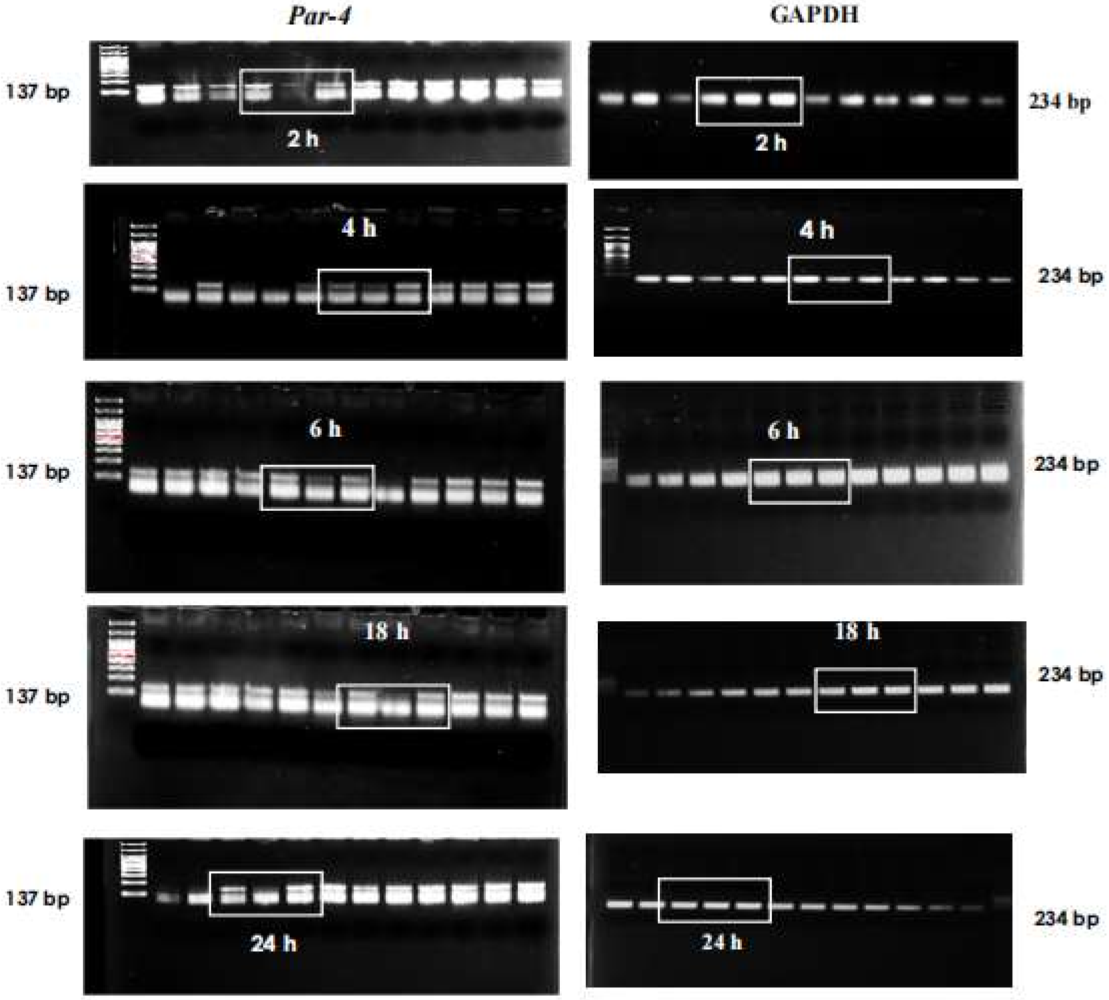

Figure S3. Raw data for agarose gel of Figure 7C

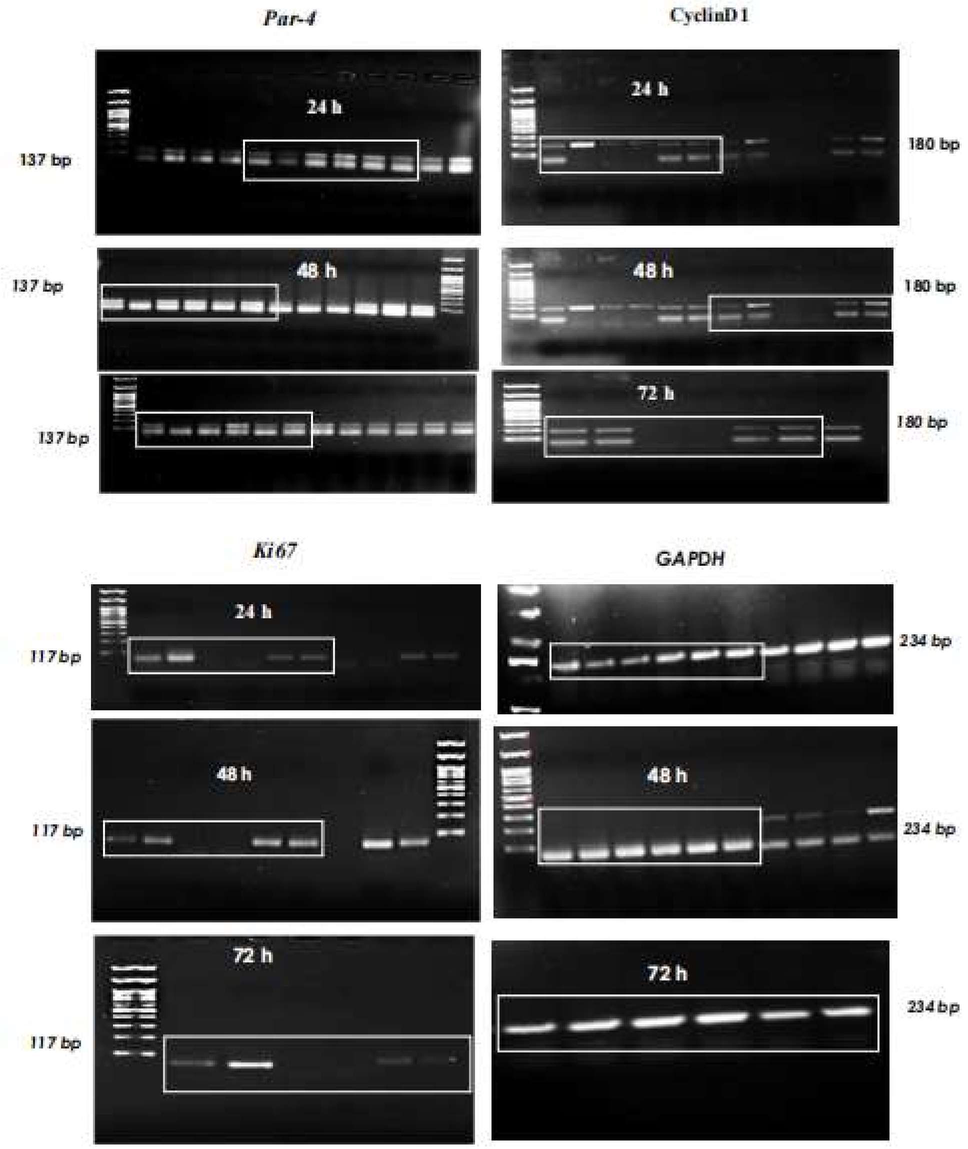

Figure S4. Raw data for agarose gel of Figure 7E

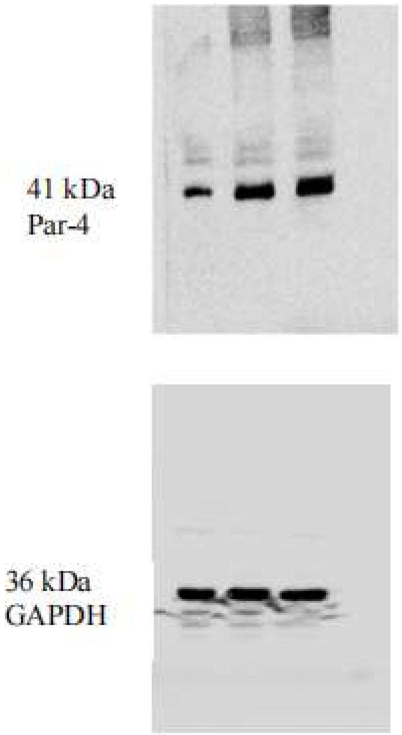

Figure S5. Raw data for immunoblot from Figure 7B

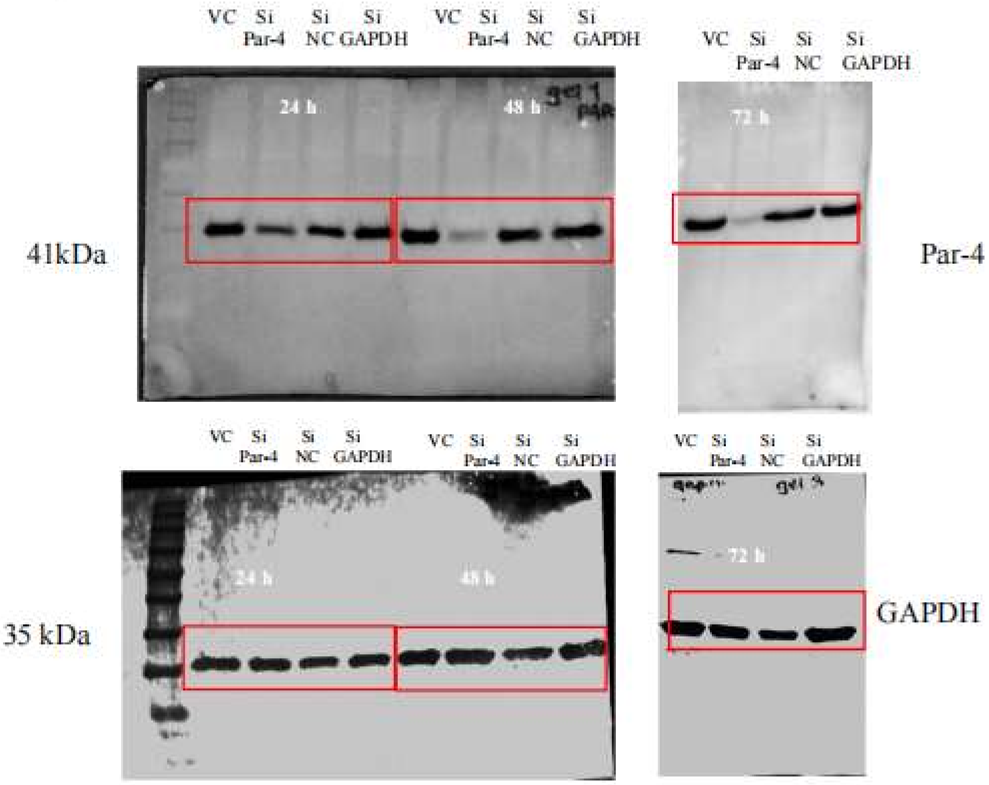

Figure S6. Raw data for immunoblot from Figure 7D

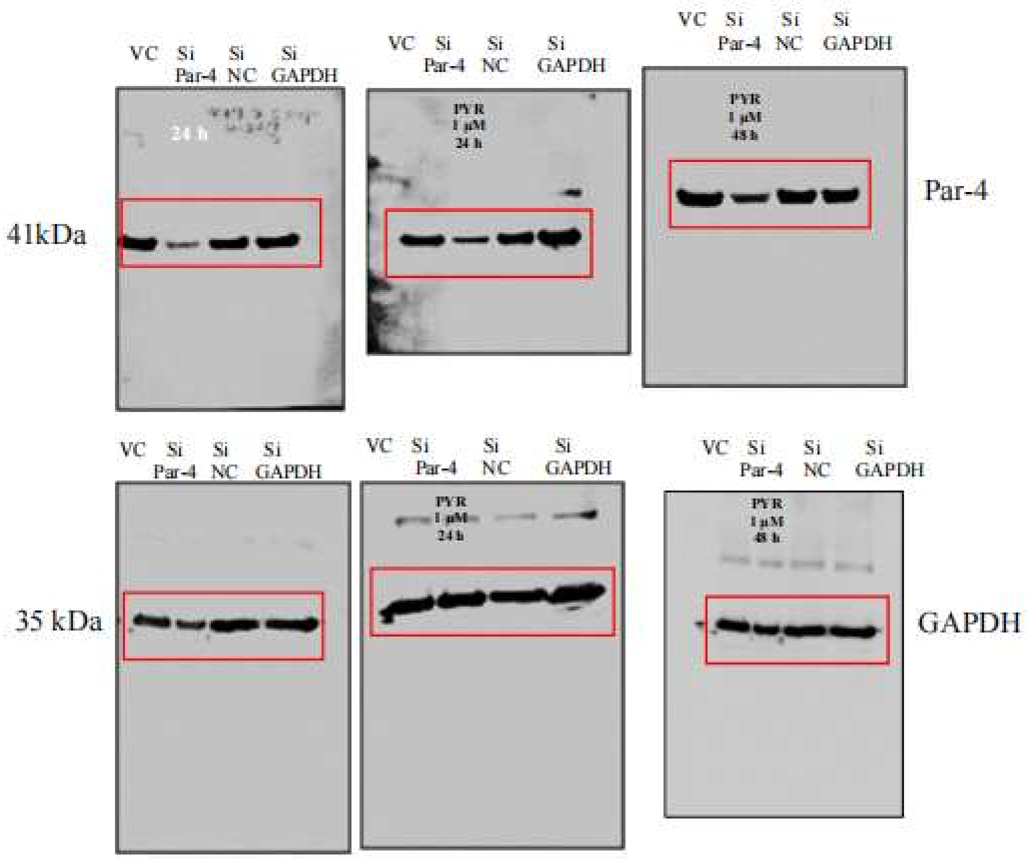

Figure S7. Raw data for immunoblot from Figure 7F

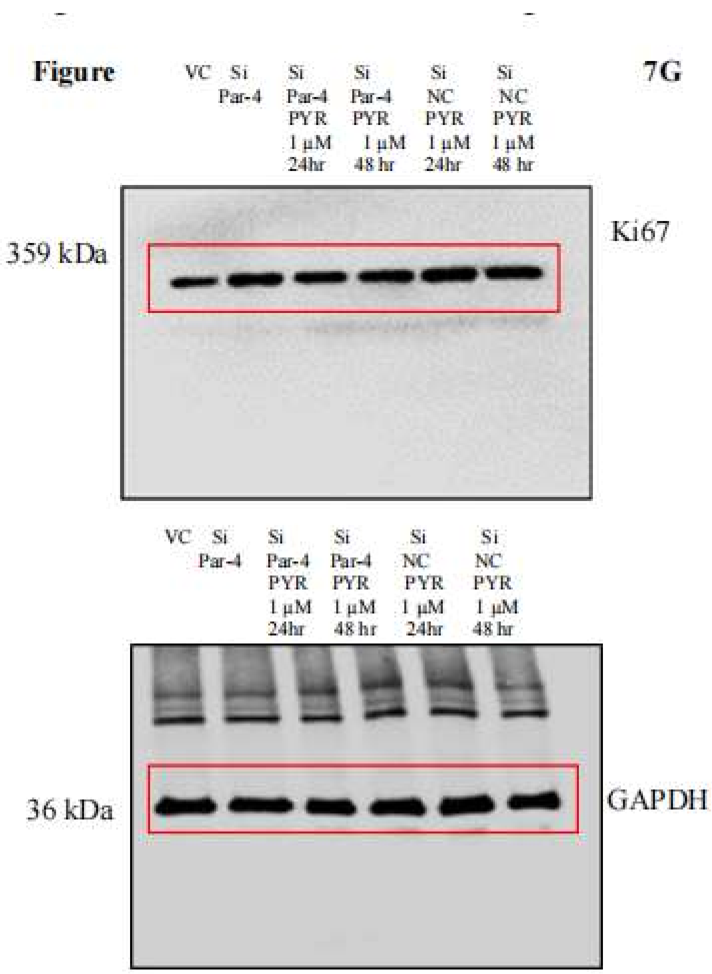

Figure S8. Raw data for immunoblot from Figure 7G

Figure 7H

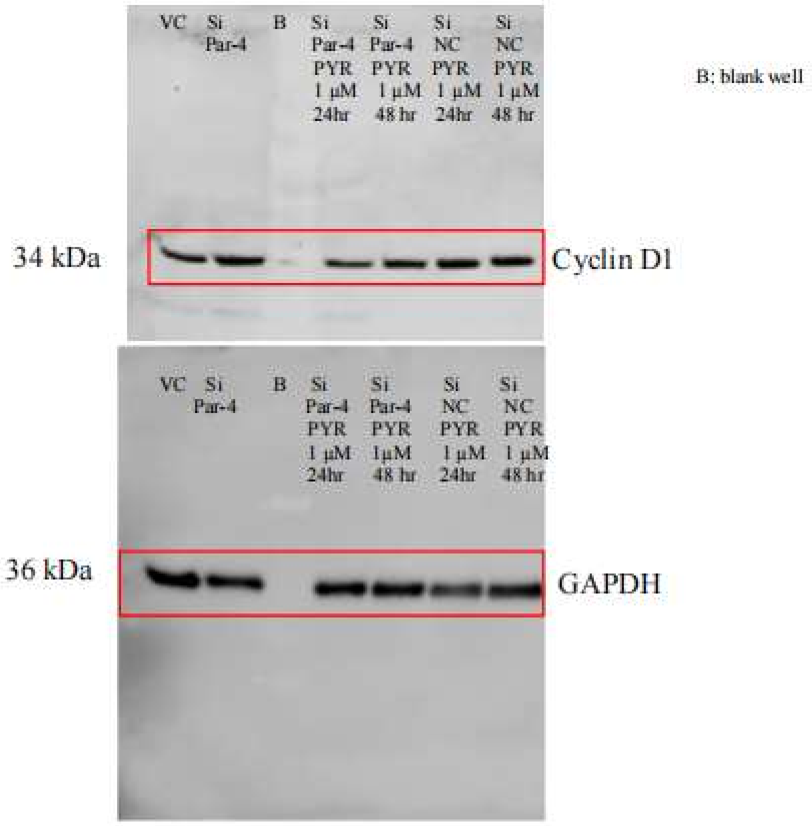

Figure S9. Raw data for immunoblot from Figure 7H

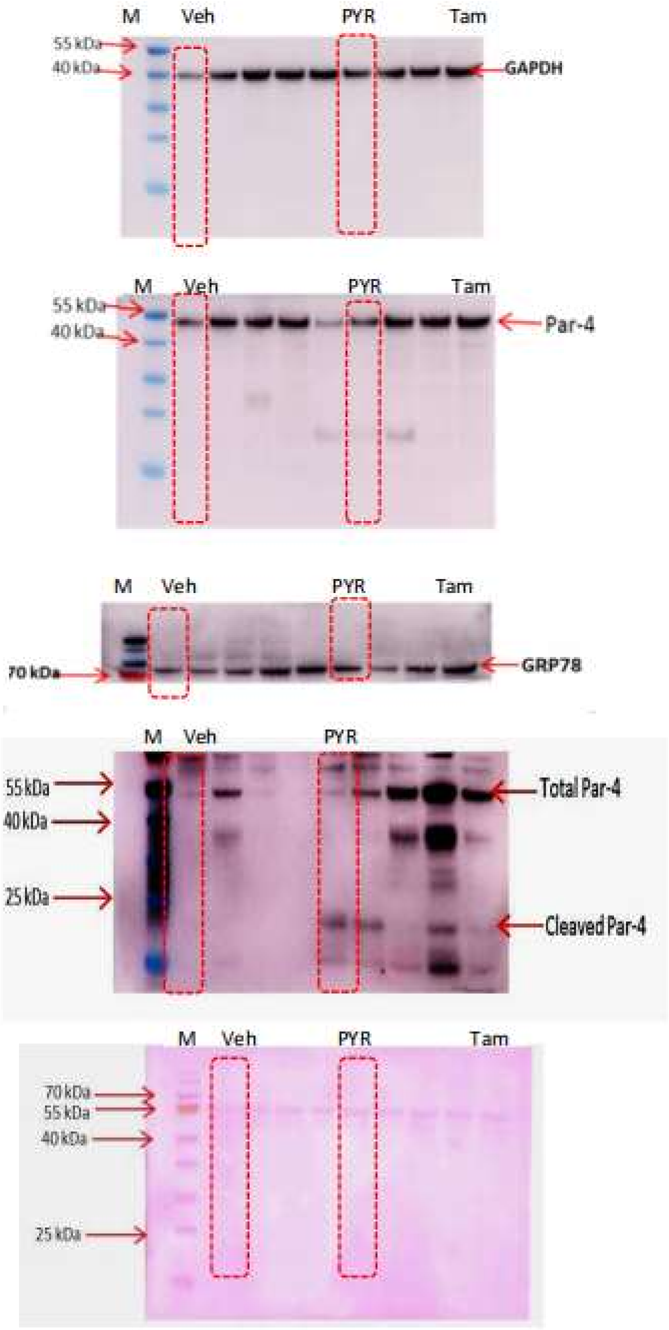

Figure S10. Raw data for immunoblot from Figure 8B **(LN-18).**

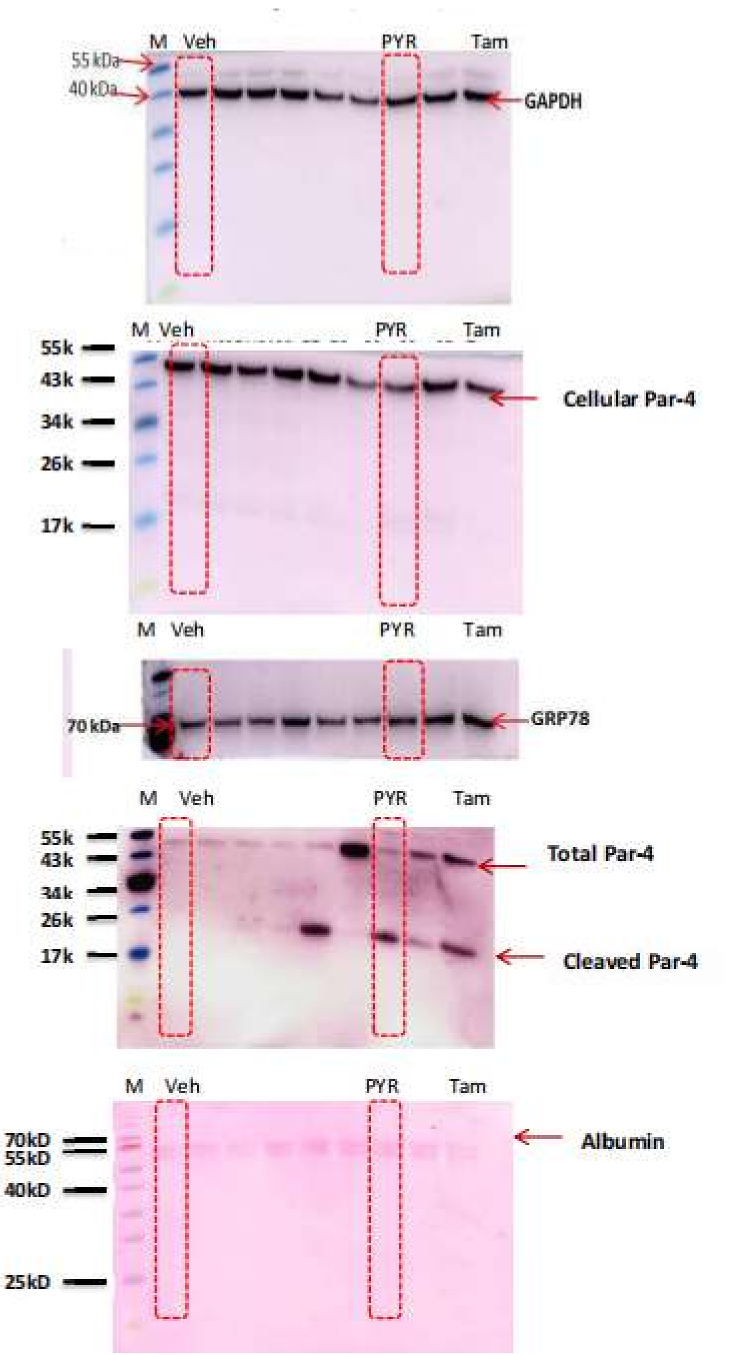

Figure S11. Raw data for immunoblot from Figure 8B **(HNGC-2)** .

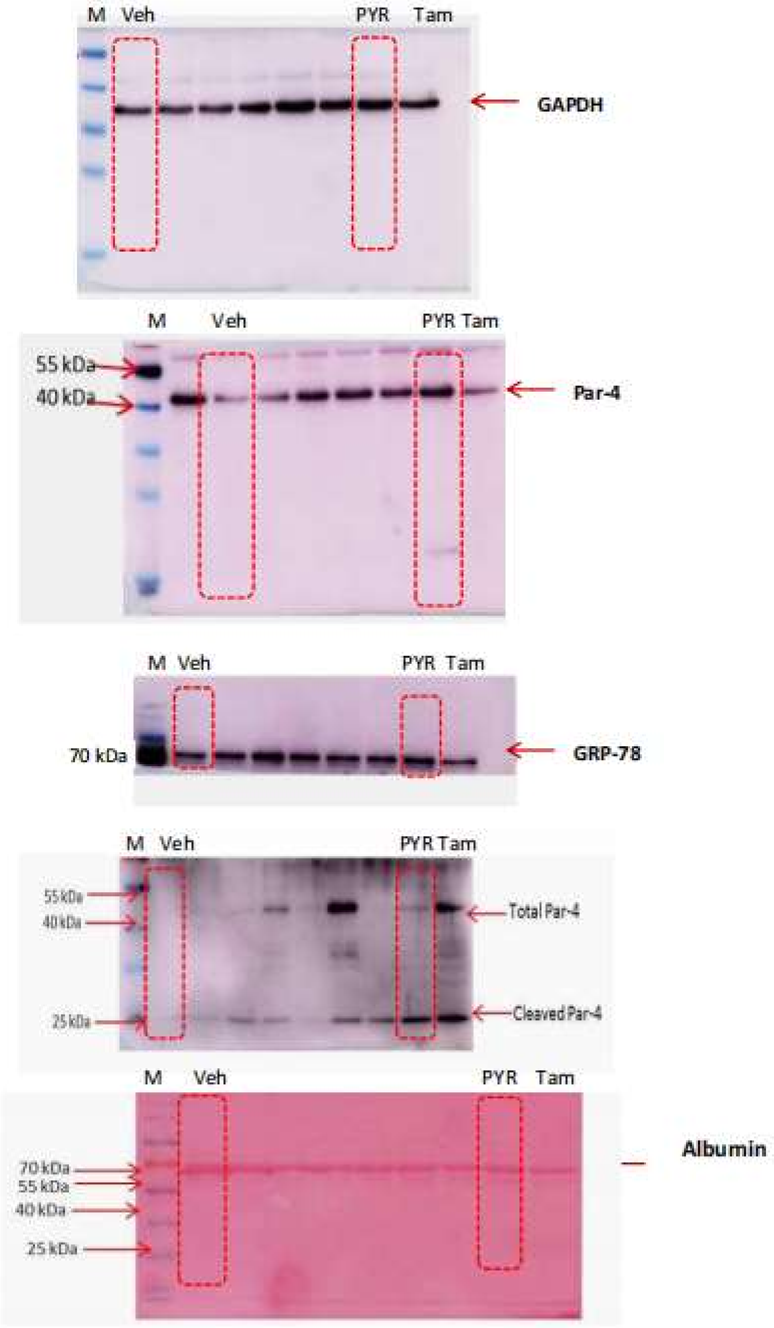

Figure S12. Raw data for immunoblot from Figure 8B **(G1 cells.**

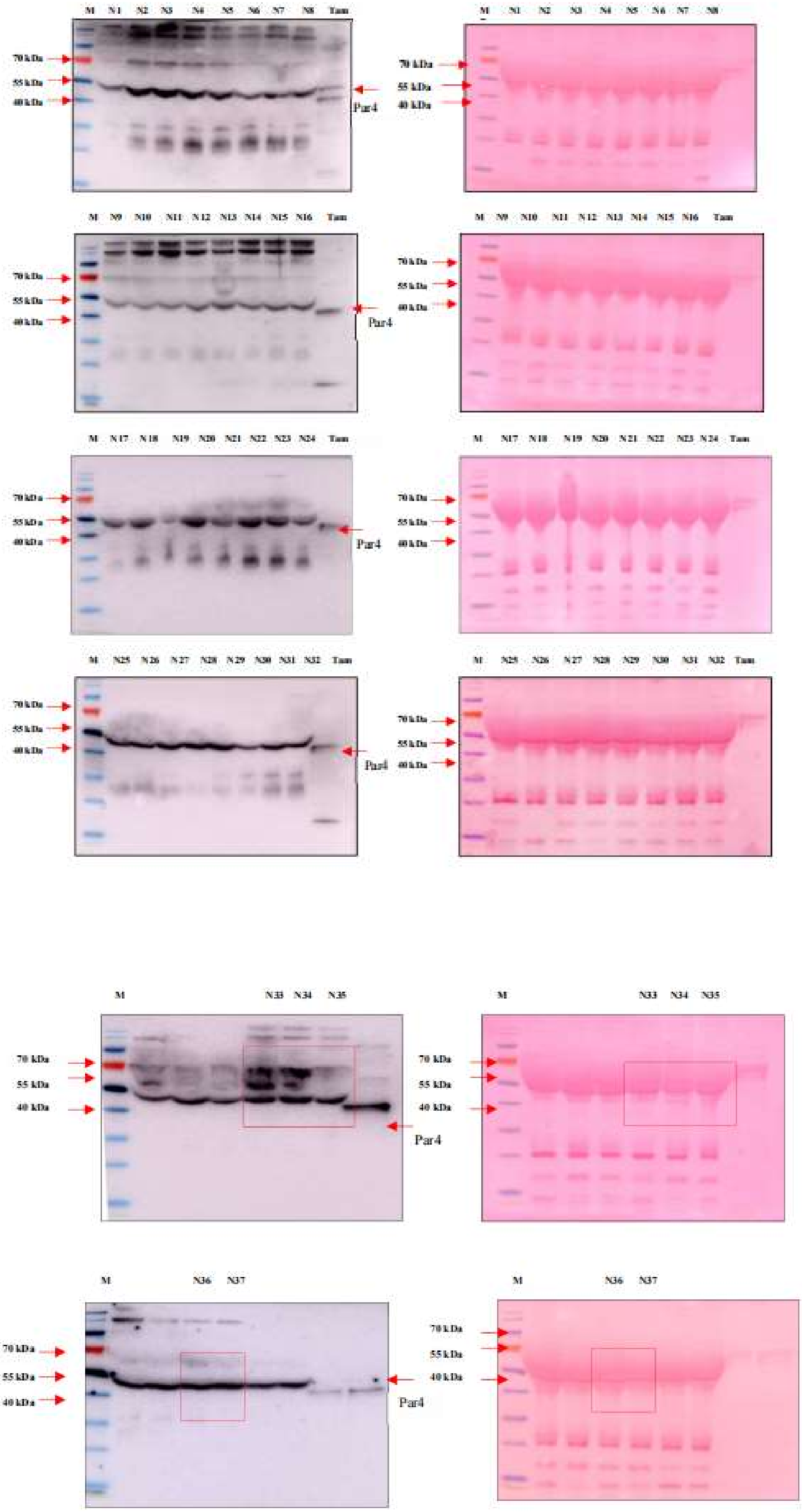

Figure S13. Raw data of immunoblot from Figure 9A

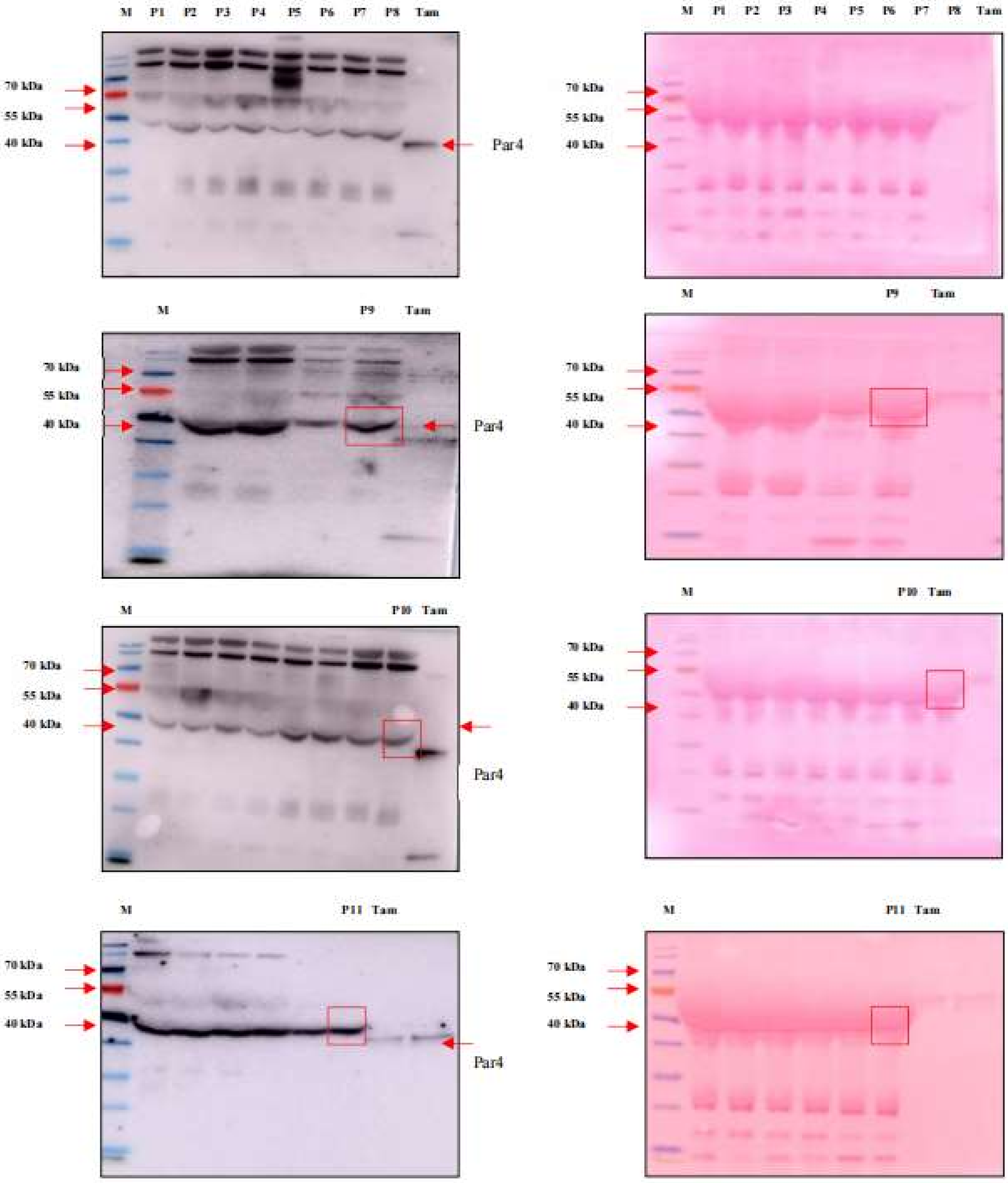

Figure S14. Raw data of immunoblot from Figure 9B
