# Supplementary material for: Prostate Apoptosis Response-4 (Par-4): A Novel Target in Pyronaridine-Induced Apoptosis in Glioblastoma (GBM) Cells
Source: Cancers (Basel). 2022 Jun 29;14(13):3198. doi: 10.3390/cancers14133198 (PMC9264948; doi:10.3390/cancers14133198)
Supplement: Supplementary file 1 [file cancers-14-03198-s001.zip › cancers-1779254-Supplementary S1.pdf]

Prostate apoptosis response-4 (Par-4): a novel target in Pyronaridine-induced apoptosis inglioblastoma (GBM) cells

Supplementary S1:

Table S1: Anti-cancer activity of PYR in various cancer types.

Cancer	Cell line	Classification	IC50 (μM)	Ref
Breast cancer	MDA-MB-231	TNBC	1.6	Villanueva PJ et al, 2018
	MDA-MB-468	TNBC	1.7	
	T47D	ER, PR +ve, Her2 -ve	9.4	
	HCC1419	ER, HER +ve, PR-ve	7	Jing Qi et al, 2012
	MCF-7	ER, PR +ve, Her2 -ve	1.6	
	MCF-7	ER, PR +ve, Her2 -ve	9.5	
	HCC70	TNBC	1.5	
Burkitt's Lymphoma	Ramos	*TP53*	2.2	Villanueva PJ et al, 2018
T-cell Lymphoma	Jurkat	*TP53, PTEN*	2	Villanueva PJ et al, 2018
	CEM	*CDKN2A*	4.6	
Pancreatic	Panc-1	*TP53*	6.5	Villanueva PJ et al, 2018
Ovarian	Ovcar-8	*TP53*	1.7	Villanueva PJ et al, 2018
	Ovcar-5	*KRAS (G12V)*	1.7	
	Ovcar-5	*KRAS (G12V)*	1.7	Jing Qi et al, 2012
	Ovcar-3	*TP53*	3.3	
	SKOV-3	*PIK3CA.2*	9.7	
	ES-2	*BRAF (V660E)*	12.9	
	PA-1	*NRas (G12D),*	15.7	
NSCLC	A549	*KRAS (G12D), CDKN2A, STK11*	3.5	Villanueva PJ et al, 2018
Melanoma	A357	*K-RAS*	2	Villanueva PJ et al, 2018
Leukemia	HL-60	CDKN2A, NRAS (Q61L), p53	1.9	Villanueva PJ et al, 2018
	K562	CDKN2A, NRAS (Q61L), p53	8.3	Jing Qi et al, 2012
Epidermal carcinoma	KB	No noted mutations	20.8	Jing Qi et al, 2012
Gastric carcinoma	BGG-823	*TP53*	14.9	Jing Qi et al, 2012
Colon	LoVo	APC, KRAS (G13D)	21.4	Jing Qi et al, 2012
Hepatocellular carcinoma	SMMZ-7721	No noted mutations	10.9	Jing Qi et al, 2012
	QGY-7703	No noted mutations	17.1	

Table S2: Histology of GBM tumor cell lines.

Tumor desig.	Model	Age	Material used	Passage	Stage at implantation
CNXF	2599L	78, M	PDX sus.	12N1	Differentiation not known
CNXF	SNB-19	47, M	NCI	16N2	Differentiation not known
CNXF	U-251	NA	NCI	17N2	Differentiation not known
CNXF	U87MG	44, F	ECACC	14N2	*PTEN, NF1(K1444M), CDKN2A, CDKN2C*
CNXF	LN-229	60, F	ATCC line	33N2	*RAD21 (Q132*) TSG, ERBB2 (L755S)*

Table S3: Mutations in GBM cells

Mutation	LN-18	LN-229	SNB-19	HNGC-2	U-251	U87MG
p53	Mutated	Mutated	Mutated	Mutated	Mutated	Wild
MGMT	High	Low	Low	NA	Low	Low
IDH	Wild	Wild	Wild	NA	Wild	Wild
PTEN	Wild	Wild	Wild	NA	Mutated	Mutated
TMZ	Resistant	Sensitive	Sensitive	NA	Sensitive	Sensitive

- *[#]HNCG-2 is patient derived Primary culture express neural stem cell marker like nestin, Vimentin and NFP-160[#]*
- *[$]G1- Primary culture derived from high grade glioma expressing neural stem cell marker like musashi, nestin and vimentin*
- *CNFX-2599L is patient derived cells (PDX) from 78-year male (#12N1) with poor differentiation*
- *NA - not available*

#Shiras A, Bhosale A, Shepal V, et al. A unique model system for tumor progression in GBM comprising two developed human neuro-epithelial cell lines with differential transforming potential and co-expressing neuronal and glial markers. Neoplasia. 2003;5(6):520-532. doi:10.1016/s1476-5586(03)80036-2

$Jagtap JC, Dawood P, Shah RD, et al. Expression and regulation of prostate apoptosis response-4 (Par-4) in human glioma stem cells in drug-induced apoptosis [published correction appears in PLoS One. 2014;9(4)

Table S4. Genetic background and characteristic of TMZ resistant and sensitive GBM cell

Properties	LN-18	U87MG
Source	65 years' patient	unknown
Gender	Male	Male
Tissue	Brain/cerebrum right temporal lobe	Brain, astrocytoma
Disease	Grade IV. GBM, Glioma	Likely glioma of CNS origin
Karyotype	n ~ 70–80, modal: 78	Hypodiploid (n ~ 44, 48%)
PTEN	Wild	Mutated
p53	Mutated	Wild
CDKN2A (p14ARF/p16INK4a)	Homozygous Deletion	Homozygous Deletion
MGMT (Promoter methylation)	Upregulated, Non-methylated	Low levels of methylation

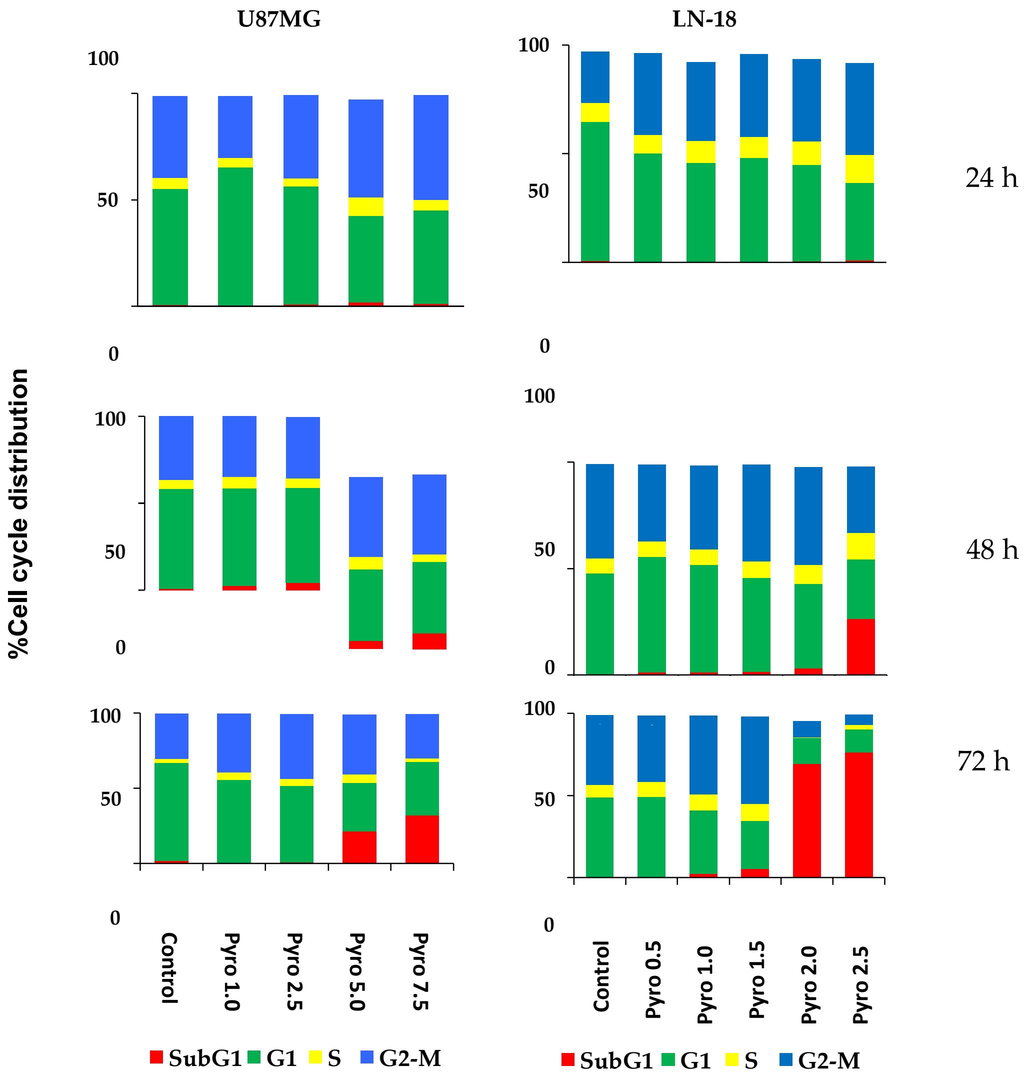

Figure S1. PYR induced cell cycle changes in GBM cell lines after 24, 48 and 72h. Changes in cell cycle in U87MG and LN-18 cells post PYR treatment at different concentration at24, 48 and 72 h. Ten thousand cells were acquired and analyzed by BD FACS-Lyric flow cytometry. Histograms represent phases of cell cycle in both GBM cells after treatment with PYR.

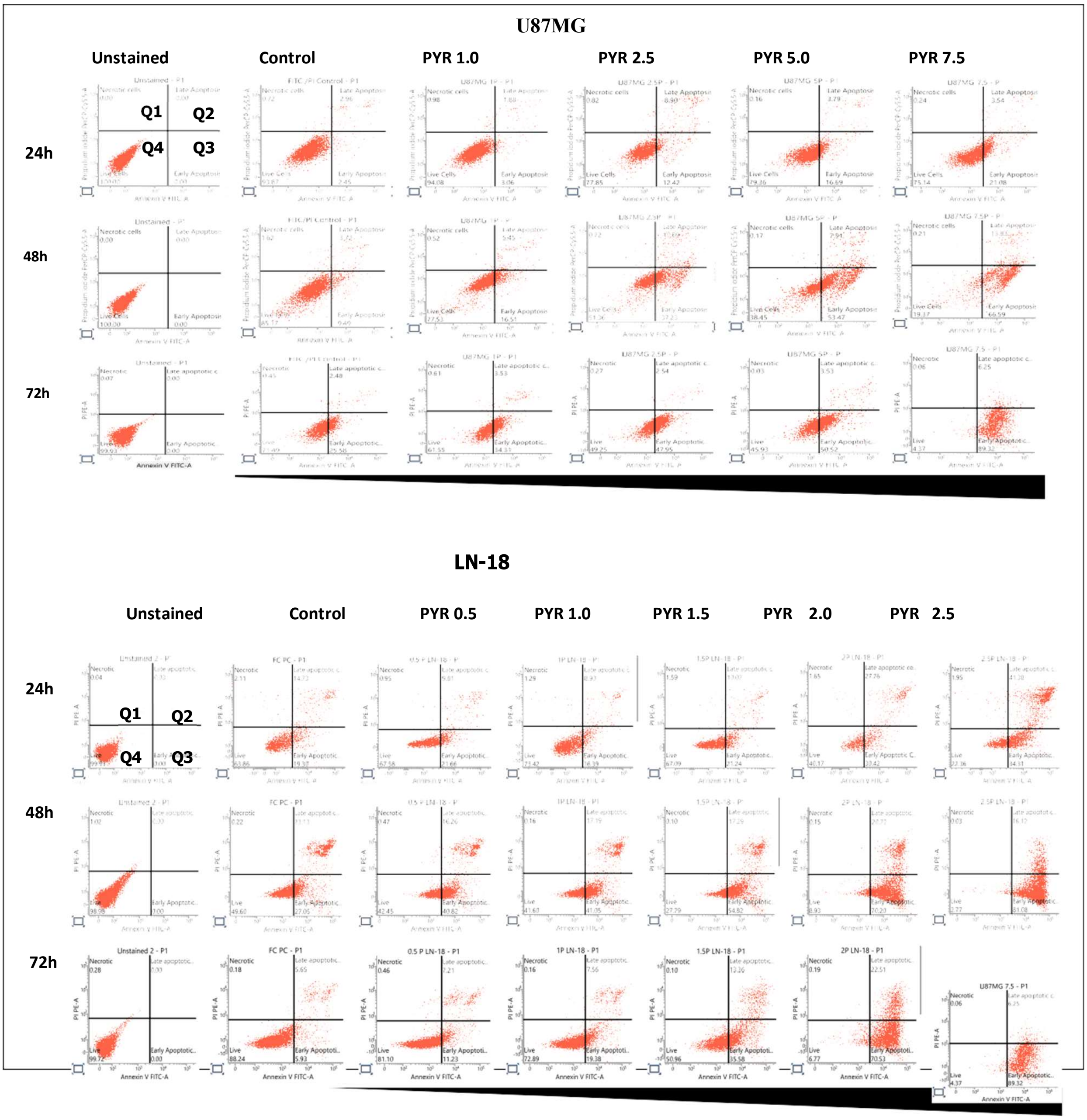

Figure S2: PYR induced Annexin V early apoptosis in GBM cells. Depicting time and dose-dependent annexin V assay in U87MG and LN-18 cells after treatment with PYR.Cells were grown in 6 well-plate and treated with different concentrations of PYR for 24, 48 and 72 h. A total 10,000 events were acquired and analysed using flow cytometry (BD FACS Lyric). Analysis of apoptosis was performed using Annexin V and PI stained cells. Four populations were indicated as non-apoptotic dead cells

(Q1), Annexin-V-FITC+/PI+ cells indicate late apoptosis cells (Q2), viable cells (Q3) and early apoptosis cells - Annexin-V-FITC+/PI− cells (Q4). The early apoptotic cells were increased due to PYR treatment.

Modelled T/C (U87MG)				
Doxo 10	0.44	0.41	0.38	0.33
Doxo 3	0.48	0.46	0.42	0.38
Doxo 1	0.43	0.43	0.55	0.37
Doxo 0.3	0.47	0.50	0.32	0.31
Doxo 0.1	0.73	0.74	0.51	0.26
Doxo 0.03	0.79	0.80	0.63	0.49
Doxo 0.01	0.80	0.81	0.81	0.50
Doxo 0.003	0.95	0.95	0.89	0.96
0	1.00	0.96	0.94	0.92
	0	PYR 0.5	PYR 1.5	PYR 2.5

Bliss - modelled T/C (U87MG)				
Doxo 10	0	0.01	0.03	0.07
Doxo 3	0	0.00	0.04	0.06
Doxo 1	0	-0.02	-0.14	0.03
Doxo 0.3	0	-0.05	0.13	0.13
Doxo 0.1	0	-0.04	0.18	0.41
Doxo 0.03	0	-0.08	0.03	0.20
Doxo 0.01	0	-0.04	0.11	0.24
Doxo 0.003	0	-0.04	0.06	-0.08
0	0	0	0	0
	0	PYR 0.5	PYR 1.5	PYR 2.5

Modelled T/C (LN-18)			
Doxo 10	0.27	0.25	0.25
Doxo 3	0.31	0.30	0.28
Doxo 1	0.32	0.27	0.28
Doxo 0.3	0.45	0.40	0.34
Doxo 0.1	0.66	0.58	0.42
Doxo 0.03	0.86	0.70	0.44
Doxo 0.01	0.79	0.73	0.53
Doxo 0.003	0.95	0.87	0.52
0	1.00	0.82	0.71
	0	PYR 0.5	PYR 1.5

Bliss - modelled T/C (LN-18)			
Doxo 10	0	-0.03	-0.06
Doxo 3	0	-0.05	-0.06
Doxo 1	0	0.00	-0.06
Doxo 0.3	0	-0.03	-0.03
Doxo 0.1	0	-0.03	0.05
Doxo 0.03	0	0.01	0.17
Doxo 0.01	0	-0.08	0.03
Doxo 0.003	0	-0.09	0.15
0	0	0	0
	0	PYR 0.5	PYR 1.5

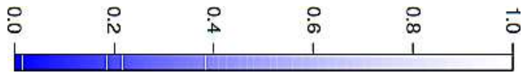

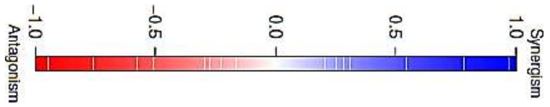

Figure S3. PYR synergises doxorubicin in GBM cells. Calculation of bliss index for combination of PYR with Doxorubicin in U87MG and LN-18 cells. Bliss index is shown as the difference of the expected T/C value (Bliss neutral) and the measured T/C value (modelled T/C) on a scale ranging from -1.0 to 1.0. Positive values (Bliss Index ≥0.15, blue) indicate synergy,negative values (Bliss Index ≤ - 0.15, red) indicate antagonism, and zero is the neutral value (white).

Oligo Name	Sequence 5' to 3'
gapdh F.P.	5' CGAGATCCCTCCAAAATCAA 3'
gapdh R.P	5' ATCCACAGTCTTCTGGGTGG 3'
par-4 F.P	5' GCCAACGAGCTCAACAACAA 3'
par-4 R.P	5' GAGCTCTTGCCCTTCTCTGG 3'
cyclin D1 F.P	5' GGCGGAGGAGAACAAACAGA 3'
cyclin D1 R.P	5' TGTGAGGCGGTAGTAGGACA 3'
ki67 F.P	5' CGACCCTACAGAGTGCTCAACAAC 3'
ki67 R.P	5' AACTGCGGTTGCTCCTTCACT 3'
Nanog F.P	5' ATCCAGCTTGTCCCCAAAG 3'
Nanog R.P	5' ATTTCATTCTCTGGTTCTGG 3'
Oct-4 F.P	5' GGTATTCAGCCAAACGACC 3'
Oct-4 R.P	5' TGATCGCTTGCCCTTCTGGC 3'
SUMO F.P	5' CCGACGAAAAGCCCAAGGAA 3'
SUMO R.P	5' TCCTCCATTTCCAATGTCGT 3'
SOX-2 F.P	5' ACAACTCGGAGATCAGCA 3'
SOX-2 R.P	5' TTAGCCTCGTCGATGAAC 3'
β-Catenin F.P	5' ATGGCTGAAGGTGACAGAGC 3'
β-Catenin R.P	5' CACCTTCCATGACAGACCCC 3'
DDX3 F.P	5' GGAGGAAGTACAGCCAGCAAAG 3'
DDX3 R.P	5' CTGCCAATGCCATCGTAATCACTC 3'
E-Cadherin F.P	5' ATGGCTGAAGGTGACAGAGC 3'

Figure S4: Primer sequences. Primer sequences used for gene expression

The profile of thermal cycling consisted of

1. Initial denaturation at 95°C for 2 min
2. Denaturation, 58°C for 50s for primer annealing
3. Extension at 72°C for 1.30s.
4. Final denaturation for 95°C for 30s

Total 35 cycles

Final extension was for 5 min. Melting curve analysis was used to determine the specific PCR products. All primers used for Real-Time PCR analysis were synthesized by Sigma.

Figure S5: qPCR conditions

Figure S6: Modulation of stem cell, hypoxia, proliferation and EMT genes markers in GBM Cells

LN-18 **U87MG**

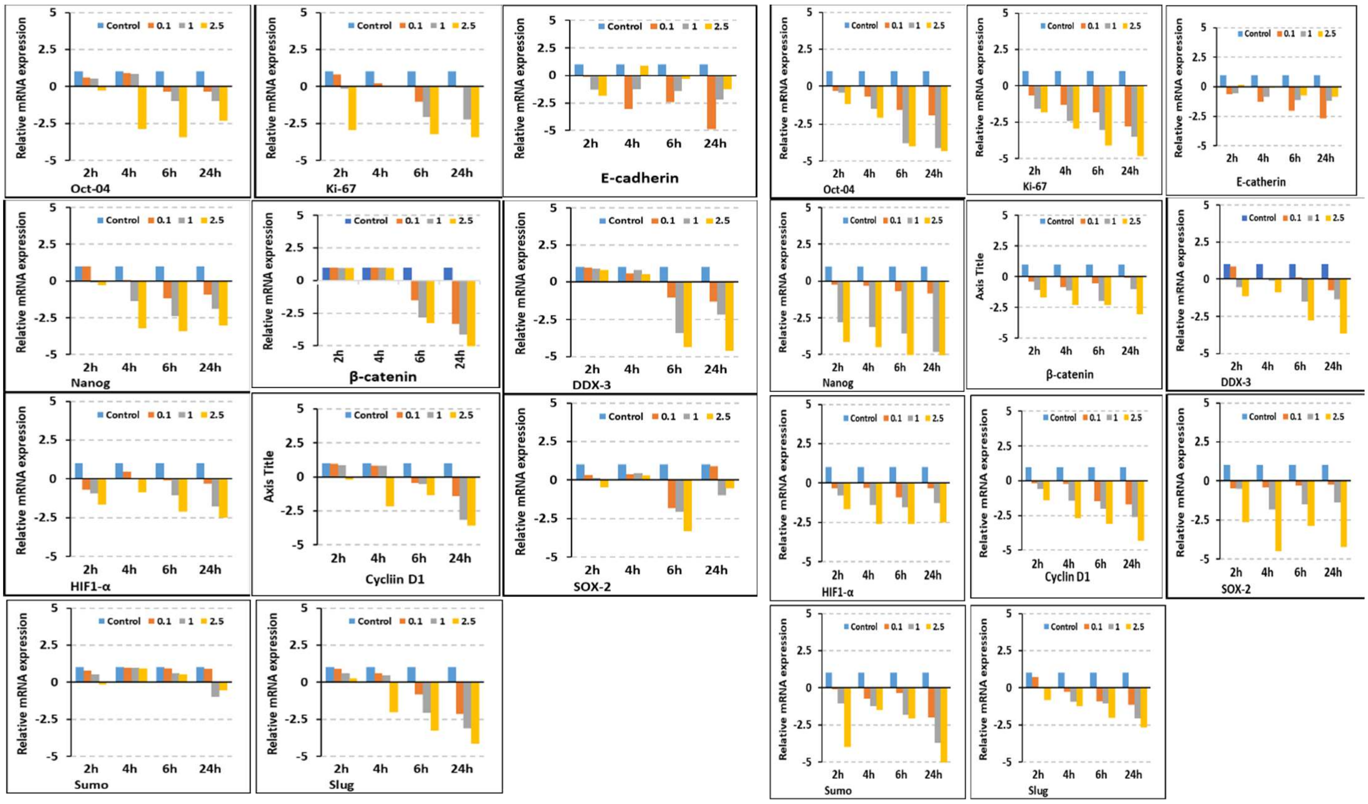

S10: Dose and time dependent modulation of gene expressions in U87MG and LN-18. The relative expression of genes was calculated with the relative ΔΔCt method, using GAPDH as housekeeping gene for normalization of data. Statistically significant ($p < 0.05$) inhibitory effect of PYR in dose dependent manner was observed for the transcripts of Oct-4, Nanog, Ki67, β-catenin, DDX3, Slug, SUMO and Cyclin D1. The significant upregulation of the transcript for E-Cadherin was noted at 24 h.

Figure S7: Online BBB predictor for PYR, TMZ and Penicilline

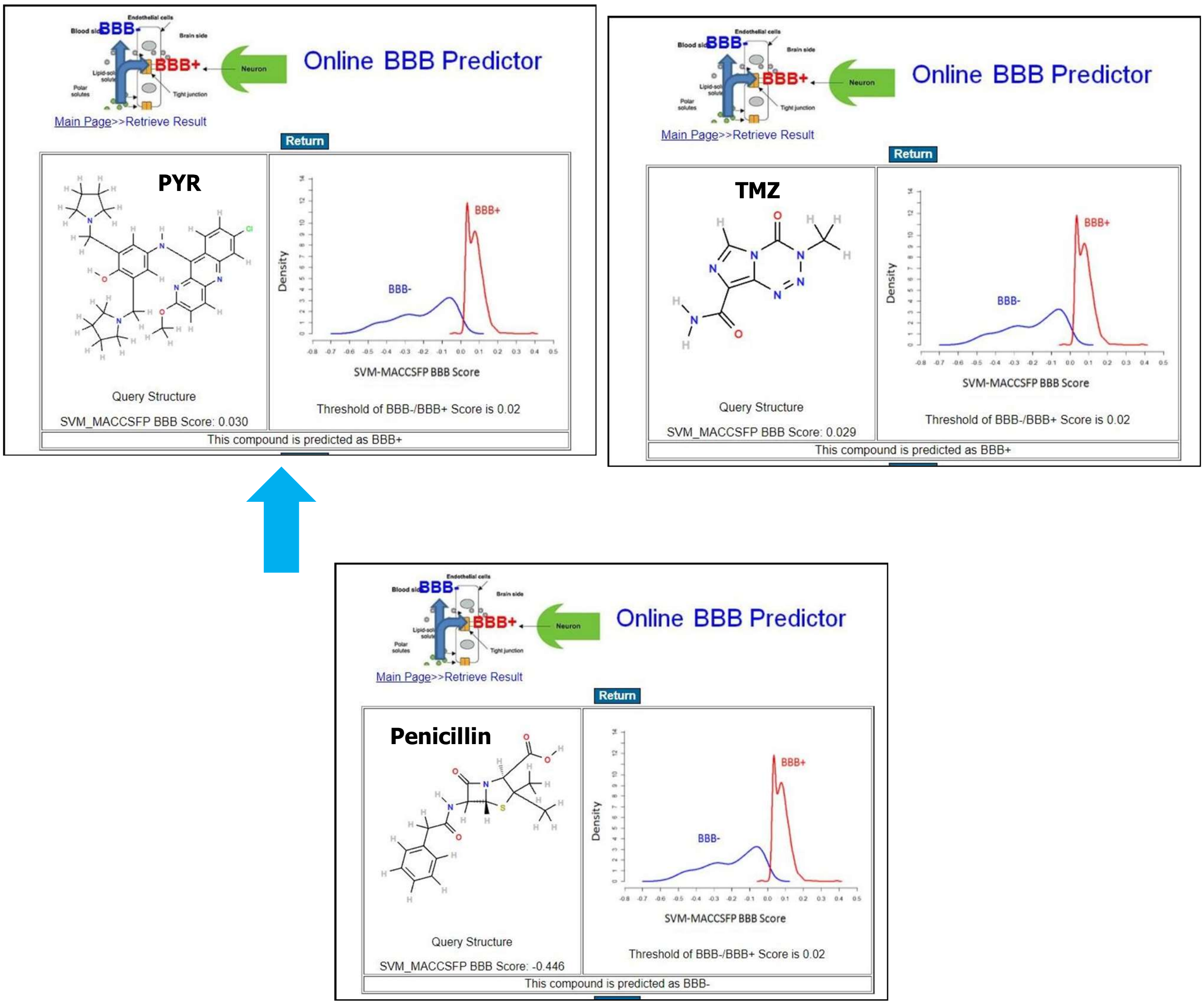

S7: BBB prediction using online BBB predictor algoritham for PYR, TMZ and Penicillin. Threshold of +BBB/-BBB was 0.03, 0.029 and – 0.0446 for PYR, TMZ (Positive control) and Penicillin (Negative control) respectively. PYR was found to be predicted as BBB+ indicating its ability to pass blood-brain barrier.
